# Working Memory Training for Schoolchildren Improves Working Memory, with No Transfer Effects on Intelligence

**DOI:** 10.3390/jintelligence5040036

**Published:** 2017-12-13

**Authors:** Dorota Żelechowska, Justyna Sarzyńska, Edward Nęcka

**Affiliations:** 1 Institute of Psychology, Adam Mickiewicz University in Poznań, 61-712 Poznań, Poland; dorota.zelechowska@gmail.com; 2 Institute of Psychology, Polish Academy of Sciences, 00-378 Warsaw, Poland; jsarzynska@swps.edu.pl; 3 Institute of Psychology, Jagiellonian University in Kraków, 31-007 Kraków, Poland

**Keywords:** cognitive training, working memory, intelligence, schoolchildren

## Abstract

Working memory contributes to many higher-order cognitive processes and predicts general cognitive skills. It is therefore important to know if its functions are trainable. In this study we investigated the malleability of working memory processes in schoolchildren whose cognitive functions are still developing. We also analyzed transfer effects to both general and specific intellectual skills. To address these issues, we examined the effectiveness of working memory training (10 training sessions) in terms of practice effects (trained tasks), near-transfer effects (working memory capacity), and far-transfer effects (psychometric intelligence). Sixty-nine children aged 8–10 participated in the study. The experimental group (42 children) participated in working memory training that intensely engaged the updating function of working memory. The training tasks, implemented as computer games, were based on the n-back and keep track paradigms. There was also an active control group (27 children). The results suggest that the experimental group improved their working memory capacity, as measured with both trained and untrained tasks. Regarding intelligence, far-transfer effects were weak and may be attributed to mere repetition of measurements. Moreover, whereas improvement in the training tasks could be observed after 15 months, the far-transfer effects disappeared in the delayed assessment.

## 1. Introduction

Are higher-order human cognitive skills malleable? If so, are short-term training interventions capable of improving these skills? This issue is now intensely debated, particularly in reference to fluid intelligence and working memory capacity [1,2,3]. The aforementioned questions have practical significance because of the widespread hope of improving our minds through deliberate practice [4]. They also have theoretical relevance, particularly from the perspective of the nature vs. nurture debate [5], as well as in relation to the issue of the cognitive underpinnings of intelligence [6]. In this paper, we present empirical evidence suggesting that children’s working memory can be improved thanks to short-term training in the form of computer games. However, such interventions can only improve working memory, with no noticeable or long-lasting effects on intelligence, understood as the general human ability to deal with novel or complex tasks [7].

Working memory (WM) is defined as the mechanism responsible for short-term storage and manipulation of information [8,9]. It is also hypothesized to involve control and regulation processes [10]. Because of its functions, WM is investigated as a possible determinant of complex cognitive processes (e.g., thinking and problem solving) or complex cognitive skills (e.g., intelligence or language proficiency). Another commonly postulated correlate of WM is academic performance, especially in the area of language and mathematics [11,12,13,14,15,16]. Consequently, it seems that attempts to improve WM might be of particular significance among schoolchildren.

A review of the literature shows that cognitive training usually leads to improvements in performance in training tasks (practice effect) or tasks based on similar procedures (near-transfer effect). Improvements in any tasks differing from the practiced ones (far-transfer effect, division by: [17]) are not that frequent, although they might have much more important implications. Near-transfer effects were found in multiple studies with adults [18,19,20,21,22,23,24,25] as well as children [26,27,28,29,30,31]. Concerning far-transfer effects, in adults they have been demonstrated as pertaining to storage of information in working memory [32,33,34,35], and executive functions such as prepotent response inhibition [18] or task switching [36,37,38]. Despite many attempts, the far-transfer effects of WM training in the area of intelligence are usually found to be insignificant [17,24,25,32,35,39,40,41,42]. Studies demonstrating significant far-transfer effects are less frequently published [20,43,44,45,46].

Far-transfer effects are more often observed in children, especially if they are developing atypically. Among children with developmental impairments, working memory training seems capable of improving the storage function of WM, as estimated by cognitive tasks [21,47,48,49,50] or by questionnaires completed by parents [51]. Similar transfer effects can sometimes be observed in relation to training of typically developing children, too. Some studies point out improvements in children’s storage mechanisms of WM [28,29,52] and executive control [52,53].

Nonetheless, there is ongoing controversy about the effectiveness of WM training in terms of enhancement of intelligence. Far-transfer effects on intelligence have been reported in children with ADHD [21,50], but other studies of similar groups failed to show corresponding results [49]. Additionally, WM training failed to show a significant influence on intelligence in studies of children with special educational needs [47,48]. The aforementioned training mostly involved span tasks that strongly engaged the WM storage mechanism. Among typically developing children, these methods did not influence intelligence [26,28,29]; however, intelligence improvements were observed after training procedures aimed at activation of executive functions [27,30,54].

Concerning the distinct aspects of working memory, the function of updating (WMU) shows the strongest correlation with both fluid and general intelligence [55]. Updating seems to be the key function in estimating the general effectiveness and capacity of working memory mechanisms [56]. Moreover, updating is the executive function that shows the highest degree of individual differences among children [57] and it may determine performance in tasks engaging both verbal and visuo-spatial working memory [16]. It seems that among typically developing children, greater improvements in the area of intelligence after WM training should be observed when training activates the updating function. This conclusion fits the trend in cognitive research described by Morrison and Chein [58], who suggest that training programs need to consist of a number of tasks that activate one particular cognitive function. Such a research strategy should allow identification of the mechanism that mediates possible far-transfer effects.

In the present study, we decided to comply with this approach. We designed training procedures that involved tasks aimed at the activation of the updating function because it is believed to mediate the relationship between working memory and intelligence. The aim of the project was to verify the effectiveness of this type of training conducted among typically developing schoolchildren. It was assumed that training would yield both near-transfer and far-transfer effects. The former should amount to the improvement of general working memory capacity and the latter should involve intelligence, both fluid and crystallized [59].

## 2. Materials and Methods

### 2.1. Participants

Sixty-nine children (32 boys) aged eight to ten (M = 8.84, SD = 0.59) participated in the study. They were recruited from two public primary schools (grades two to four) in the Lubuskie Province of Poland. Participation in the study was voluntary. Parents decided whether to sign their child up for memory training (experimental group), or for thinking and problem solving training (control group). Such a solution was the only way to win their approval and cooperation. The experimental group consisted of 42 participants (22 boys) aged eight to nine (M = 8.79, SD = 0.42), and the control group included 27 participants (10 boys) aged eight to ten (M = 8.93, SD = 0.78).

### 2.2. Materials: Transfer Tasks

#### 2.2.1. Raven’s Progressive Matrices (RPM)

We used the Polish adaptation [60] of the standard version of *Raven’s Progressive Matrices* [61]; this tool is regarded to be a good measure of fluid intelligence. We used the test in the classic and parallel forms.

#### 2.2.2 .Wechsler Intelligence Scale for Children—Revised (WISC-R)

We used the Polish adaptation [62] of the “Wechsler Intelligence Scale for Children—Revised” [63]. This tool estimates general intelligence manifested through verbal and nonverbal tasks. The analyses included raw scores from the entire test (full scale, range 0–426 points), the Verbal Subscale (0–171 points), the Nonverbal Subscale (0–255 points), as well as from particular subtests. The subtests included five verbal tasks: *Information* (0–29 points), *Similarities* (0–32 points) *Arithmetic* (0–18 points), *Vocabulary* (0–64 points), and *Digit Span* (0–28 points). There were also five nonverbal tasks: *Picture Completion* (0–26 points), *Picture Arrangement* (0–44 points), *Block Design* (0–44 points), *Object Assembly* (0–48 points), and *Coding* (0–93 points).

#### 2.2.3. OSPAN

General working memory capacity was estimated using a version of OSPAN [64,65]. The solved equations were displayed on a screen and participants were asked to decide if the solution was correct or incorrect. Brief feedback appeared after every incorrect answer. After each equation, a word was displayed for 1600 milliseconds. There were two to five equation–word pairs in one set. After the last word in each set, the computer displayed a request to repeat all memorized words in the order of their presentation. The researcher wrote down the words spoken by the child. The task was comprised of 20 sets, five of each type, consisting of two, three, four or five equation–word pairs. The procedure was preceded by an instruction and a rehearsal consisting of four sets (one of each type). Each of the presented words consisted of five letters and had a frequency of appearance comparable to that of natural language. The equations included addition and subtraction of digits ranging from 1 to 9 (e.g., “4 + 2 = 6”). Half of the equations were correct and half were incorrect. The incorrect solutions differed from the correct ones only by 1. Equations were chosen randomly from separate sets for the rehearsal stage (14 equations) and the main stage (100 equations); each equation was displayed only once during the whole procedure. Similar rules applied to the display of words. The order of sets of different sizes was also random. Consequently, the set to be recalled was different each time so the task could be used multiple times. The index score was computed as the sum of points collected by the child in the whole task. The points were awarded only for trials in which the child properly identified the equation and recalled the set of accompanying words. A maximum of 20 points could be collected.

### 2.3. Materials: Training Tasks

The training tasks resembled children’s computer games that require speedy responses. The trainings were adaptive: the tasks’ level of difficulty changed according to the player’s competence. Each game started from the most basic level, but soon the level of difficulty was adjusted to the player’s changing level of performance. If the ratio of correct answers was over 90% in a given attempt, the difficulty of the tasks in the next attempt increased. If this ratio was between 50% and 90%, the difficulty stayed fixed; if it fell below 50%, the difficulty in the following attempt decreased. Training tasks were aimed to engage the updating function of working memory. These tasks were based on the keep track paradigm [56,66] and a modified version of the n-back paradigm [67,68]. Two games, *Sausage Dog* and the *Big Tidy-up*, were based on the keep track paradigm. Two other games, *Gotcha!* and *Zoo*, were prepared according to the n-back procedure. There were also two training tasks for the control group. These tasks were supposed to engage working memory processes to the least possible extent. The Appendix includes detailed descriptions of all the training tasks.

### 2.4. Procedure

#### 2.4.1. Initial Psychometric Testing

In the two weeks before initiation of the experimental treatment, we assessed the children’s intelligence and working memory capacity. WISC-R testing of intelligence was conducted individually during two 45-min meetings. The standard version of *Raven’s Progressive Matrices* was administered in groups consisting of five to 10 children and took ca. 30 min (the standard version has no time limit). Half of the children filled in the classic form of the standard version and half the parallel form. The computerized OSPAN test was conducted individually and lasted from 25 to 45 min. The participants took no more than one test a day.

#### 2.4.2. Training

The training proper consisted of 10 meetings lasting ca. 40 min each, plus the introductory session, the pre-test session, and the post-test session. There were three to five training sessions per week. The meetings took place in computer labs. Four to nine children participated in each meeting. Every child used a separate computer and headphones. During the introductory meeting the children were acquainted with the training methods and tried out the game at the easiest level. During the second (pre-test) and last (post-test) session the training tasks were set to a medium difficulty level, so as to estimate improvement. We conducted the tests during the second session rather than the first in order to avoid situations in which the children showed improvement only because they had failed to learn the principles of a given tasks at the initial meeting. During each training session, children from the experimental group played two out of four games designed for them, one for each procedure. The games alternated every second session. Children from the control group played both games during every session, which took 15–20 min per game, that is, 30–40 min altogether.

#### 2.4.3. Second Psychometric Testing

The testing phase took place within two weeks of completion of the training. The procedure was analogous to the one adopted in the initial testing. Children solved versions (classic or parallel) of *Raven’s Progressive Matrices* that were different than the ones they got in the initial phase of testing.

#### 2.4.4. Delayed Testing

This testing session took place 15 months after completion of the training. Thirty-one participants (10 boys) were available for participation in the delayed testing, 17 from the experimental group (six boys) and 14 from control group (four boys). For technical reasons, we shortened the battery of assessment tools used in the third measurement. As for near-transfer effects, we assessed the delayed outcomes with only the two training tasks that had produced quite a strong improvement in the second testing (see the Results section): *Gotcha!* and *Zoo*. Tasks were set to a medium level of difficulty. We also checked the delayed outcomes in reference to *Raven’s Progressive Matrices* and three subtests from the WISC-R: *Similarities*, *Vocabulary*, and *Digit span.*

### 2.5. Incentive System

After the first psychometric session, every child obtained a special “Participation sheet” and a pin with the project’s name. In this way we tried to win children’s engagement in the project activities. The “Participation sheet” included a timetable with dates of training sessions and blank spaces to be filled in with the child’s scores on subsequent games. After each meeting, the child could put a special sticker next to the date of the session that had been just completed. The child got a small gift every three stickers. Children from both the experimental and control group were provided with educational toys and our working memory training games for participation in the whole project. These gifts were delivered after all participants completed the post-test measurements. The games for the control group were set to initial levels so children could properly start the training on their own. In the case of children who had previously attended our working memory training, games were set to levels that enabled participants to continue the training on the right level of difficulty. Additionally, children who achieved the best results and the greatest improvement obtained coupons that could be exchanged for games or educational toys. Moreover, after the first meeting, when children had got accustomed to the training methods, we introduced additional rules to encourage the children to be quiet and focused during the sessions. At the beginning of each meeting, every child got five “word tokens.” A rule was introduced that each disruptive behavior cost one token. A child left with no tokens at the end of the session would get only half a sticker, which would not stop them from completing the program, but would delay getting the gift for three stickers. The children who retained all tokens received an additional “good manners sticker,” which allowed them to receive additional small gifts (similar to those received for three stickers). The children stated that the system was clear and their evaluations of the system were positive. None of the participants lost more than three tokens during any of the sessions.

## 3. Results

Firstly, we conducted a mixed design two-factor analysis of variance (ANOVA): two groups (between-person, control vs. experimental) × 2 testing time (within-person, pre-test vs. post-test). We analyzed main effects and interactions. Additional analyses were performed in order to check whether any statistically significant improvement could be detected in each group. In order to estimate the training-related improvement of performance, we created an additional variable that was computed as the difference between the second score and the first score. Secondly, we carried out a Student’s *t*-test for independent samples in order to estimate whether the groups differed in the extent of training-related improvement. Exploratory data analyses allowed us to detect that, for some variables, there were deviations from normal distributions or lack of homogeneity of variance. We decided to use the parametric approach and analyze the data using non-parametric methods (Wilcoxon signed-rank test for dependent samples), if necessary. The results of non-parametric tests matched the parametric ones, so we decided to report only the latter.

### 3.1. Practice Effects

Table 1 reports descriptive statistics of performance in the training tasks. ANOVA revealed that in the *Sausage Dog* task there was a statistically significant effect of interaction between independent variables, group, and testing time, *F*(1.67) = 30.23; *p* < 0.001; *eta*^2^ = 0.31. The experimental group did better at post-test (*p* < 0.001), but there were no differences between groups in the pre-test (*p* = 0.39). A significant improvement between the first and the second testing time took place in the experimental group (*p* < 0.001), but not in the control group (*p* = 0.1). Likewise, in the *Big Tidy-up* task the interaction effect was significant: *F*(1.67) = 6.23; *p* < 0.05; *eta*^2^ = 0.09. However, the groups differed not only at the second testing (*p* < 0.001), but also at the initial one (*p* < 0.05). In both conditions, the experimental group did better than the control one. However, we observed a statistically significant improvement in task performance only in the experimental group (*p* < 0.001), and not in the control one (*p* = 0.10). In the *Gotcha!* task the interaction effect between the group and the testing time was statistically significant: *F*(1.67) = 38.78; *p* < 0.001; *eta*^2^ = 0.38. Groups differed in task performance at the second testing (*p* < 0.001), with the experimental group doing better than the control one. However, we found no difference between the groups at initial testing (*p* = 0.11). We also observed a statistically significant improvement in task performance (*p* < 0.001) in the experimental group, but not in the control one (*p* = 0.53). Finally, in the *Zoo* task, we observed an interaction effect between the group and the testing time: *F* (1.67) = 14.28; *p* < 0.001; *eta*^2^ = 0.18. The experimental group did better in the post-test (*p* < 0.001), while the groups did not differ at pre-test (*p* = 0.62). Only the experimental group improved their task performance between two measurements (*p* < 0.001); the control group did not (*p* = 1.00).

### 3.2. Near-Transfer Effects: Working Memory

One person from the experimental group was excluded from the analyses of the change in the OSPAN task performance because the digital file containing the scores turned out to be incomplete. As for the near-transfer effects, we found a significant interaction between group and testing time, *F*(1.66) = 33.82; *p* < 0.001; *eta*^2^ = 0.34. Further analyses indicated that the groups differed only in the post-test, in which the experimental group performed better than the control group (*p* < 0.001), whereas in the pre-test the performance was similar in both groups (*p* = 0.54). The analyses also revealed that in the experimental group there was a significant training-related improvement in the OSPAN task (*p* < 0.001), whereas in the control group a small decline in performance was observed (*p* < 0.05). In additional analyses, we determined that the aforementioned pattern of results occurred at each level of difficulty of the OSPAN task, but only in the case of the experimental group. Regarding the control group, there were no improvements and a small decline could be observed only for the simplest version of the OSPAN task; this effect verged on statistical significance (*p* = 0.055), so we suggest it was incidental. The details are provided in Table 2.

### 3.3. Far-Transfer Effects: Intelligence

#### 3.3.1. Raven’s Progressive Matrices

The interaction between group and testing time did not reach the level of statistical significance, *F*(1.65) = 1; *p* = 0.32; *eta*^2^ = 0.015, thus suggesting a lack of any training-related improvement. However, the contrast analysis revealed a statistically significant improvement in performance (i.e., the contrast between pre-test and post-test) in the experimental group (M1 = 35.17, SD = 8.82, M2 = 36.88, SD = 8.67, *p* < 0.05), but not in the control group (M1 = 27.54, SD = 7.51, M2 = 28, SD = 8.53, *p* = 0.64); this may suggest that some training effects could occur. In order to obtain further evidence, we subtracted Raven scores in the first measurement from the scores in the second measurement and conducted a Student’s *t*-test on this artificial variable. It appeared that the groups did not differ in terms of performance improvement *t*(65) = 1.16; *p* = 0.27, Hedges’ *g* = 0.14. The analysis of particular test conditions (series A to E of Raven’ matrices) yielded similar results, thus suggesting a lack of any training-related effects.

These discrepancies might have been a result of the preexisting differences between the experimental and control groups, whose average Raven scores differed in both the pre-test (*p* < 0.005) and post-test (*p* < 0.001). These differences could have resulted from lack of randomization, although random assignment to the training and control group does not automatically prevent preexisting differences in the pre-test. However, possible biases due to preexisting differences between the groups motivated us to conduct additional analyses. First of all, children from the experimental group were divided into two subgroups based on the initial performance in the RPM, with the median of the experimental group (Me = 37.00) serving as the cutoff point. Because there were as many as 42 children in the experimental group, we obtained enough cases after the split. Next, we analyzed the interaction effect between the time of measurement and three groups (i.e., control, experimental with better initial performance, and experimental with worse initial performance). This interaction did not reach statistical significance (*F*(2.64) = 1.56; *p* = 0.22; *eta*^2^ = 0.045). Specifically, the control group and the “lower” experimental group (M1 = 28.10, SD = 6.36, M2 = 30.95, SD = 7) neither differed statistically in the pre-test, nor showed any differences concerning the training-related improvement in the RPM scores. Altogether, we believe that these results do not support the hypothesis that WM training improved fluid intelligence, as measured with Raven’s matrices.

#### 3.3.2. Wechsler Intelligence Scale for Children—Revised (WISC-R)

Table 3 shows descriptive statistics pertaining to the results obtained by the two groups in the WISC-R test. Regarding the full scale, we found a significant interaction effect of group and time of measurement, *F*(1.67) = 47.78; *p* < 0.001; *eta*^2^ = 0.42. The groups differed at post-test (*p* < 0.001), in which the experimental group did better. There were no significant group differences at pre-test (*p* = 0.11). Improvement in performance between the first and the second testing took place in both groups (both at *p* < 0.001). Since greater improvement was expected in the experimental group, we decided to create an artificial variable expressing the change in performance between the first and the second testing. The student’s *t*-test for independent samples showed that the performance improvement was greater in the experimental group (M = 39.81, SD = 15) than in the control group (M = 16.15, SD = 11.80), *t*(67) = 6.91; *p* < 0.001). Hedges’ *d* = 1.71 indicated a substantial effect size.

Further analyses revealed that the effect of interaction between the group and the testing time was statistically significant for both the Verbal Scale (*F*(1.67) = 74.66; *p* < 0.001; *eta*^2^ = 0.53) and the Nonverbal Scale (*F*(1.67) = 10.77; *p* < 0.005; *eta*^2^ = 0.14). For the Verbal Scale, a statistically significant improvement was found only in the experimental group (*p* < 0.001), but not in the control group (*p* = 0.93). As regards the Nonverbal Scale, the experimental group surpassed the control group both after (*p* < 0.001) and before the training (*p* < 0.05). We expected a greater improvement in the experimental group, so we conducted a Student’s *t*-test for independent samples. Indeed, improvement in the control group (M = 16.04; SD = 11.20) was smaller than in the experimental group (M = 25.17; SD = 11.33), *t*(67) = 3.28; *p* < 0.005, and Hedges’ *g* = 0.81 indicated that the effect size was substantial.

**Figure 1:**
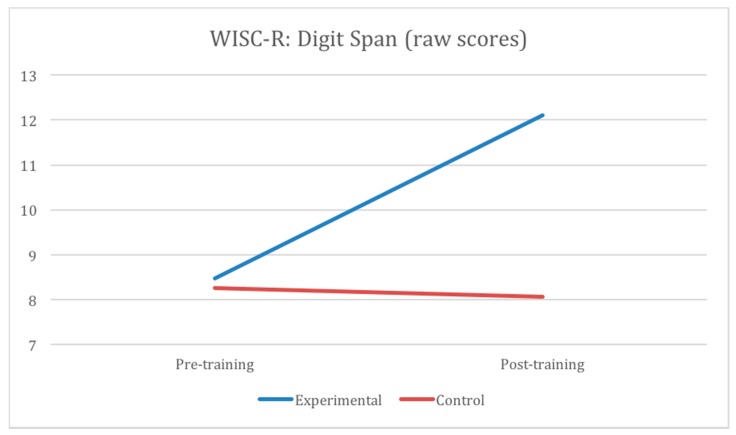
Mean raw scores of the WISC-R *Digit Span* subtest obtained by two groups in pre-test and post-test.

The aforementioned analyses might suggest that there was a significant training-related improvement of general intelligence, as measured with WISC-R full-scale and both subscales. However, the detailed analyses of 10 subtests revealed that the improvement occurred mainly in some subtests from the Verbal Scale. The significant interaction effect of group and testing time was observed for the following subtests: *Similarities* (*F*(1.67) = 24.69, *p* < 0.001, *eta*^2^ = 0.27), *Arithmetic* (*F*(1.67) = 9.24, *p* < 0.005, *eta*^2^ = 0.12), *Vocabulary* (*F*(1.67) = 27.78, *p* < 0.001, *eta*^2^ = 0.29), and *Digit Span* (*F*(1.67) = 50.12, *p* < 0.001, *eta*^2^ = 0.43). The interaction effect did not reach statistical significance for the *Information* subtest (*F*(1.67) = 1.55; *p* = 0.22; *eta*^2^ = 0.23). The strongest of these effects (Digit span) is illustrated in Figure 1. In the *Similarities, Arithmetic, Digit span* and *Information* there were no differences between groups at pre-test, but in post-test the experimental group always got better scores. Additionally, in all these tests we observed an improvement in test performance after training in the experimental group; in the control group there was only an improvement in *Information*.

For the Nonverbal Scale, the effect of interaction between the group and the measurement time was significant only in case of two subtests: *Coding* (*F*(1.67) = 5.99; *p* < 0.05; *eta*^2^ = 0.08) and *Block Design* (*F*(1.67) = 4.24; *p* < 0.05; *eta*^2^ = 0.06). Although these effects were very weak in terms of effect sizes, we subjected them to further analyses. For the *Coding* subtest, it appeared that the experimental group differed from the control group only in the second testing (experimental: M = 47.21; SD = 9.06, control: M = 41.11; SD = 8.98; *p* < 0.01), but not in the initial testing (experimental: M = 38.19; SD = 7.46, control: M = 36.11; SD = 8.50; *p* = 0.29). Both groups improved their scores between measurements, but the experimental group improved more (M = 9.02; SD = 6.81) than the control group (M = 5.00, SD = 6.42; *t*(67) = 2.45; *p* < 0.05). The analyses of performance in the *Block Design* indicated that the experimental group already surpassed the control group in the pre-test (experimental: M = 21.31; SD = 5.13, control: M = 18.59; SD = 4.03; *p* < 0.05) and the post-test (experimental: M= 24.98, SD = 5.02; control: M = 20.37; SD = 3.85; *p* < 0.001). Both groups improved their scores (*t*(67) = 2.06; *p* < 0.05), but the experimental group improved more (M = 3.67, SD = 4.23, *p* < 0.001) than the control (M = 1.78; SD = 2.71, *p* < 0.05). For the remaining subtests of the Nonverbal Scale, the interaction effect did not reach statistical significance: *Picture Completion* (*F*(1.67) = 1.19; *p* = 0.28; *eta*^2^ = 0.02), *Picture Arrangement* (*F*(1.67) = 1.24; *p* = 0.27; *eta*^2^ = 0.02), *Object Assembly* (*F*(1.67) = 0.84; *p* = 0.36; *eta*^2^ = 0.01). As we can see, subtests pertaining to the Nonverbal Scale showed either insignificant or rather faint improvement in the second measurement, compared to the first one.

Since the experimental and control group differed in the pre-test in *Block Design*, *Picture Arrangement*, and *Object Assembly*, we decided to conduct additional analyses. Analogically to the analyses referring to the RPM scores, children from the experimental group were divided into sub-groups on the basis of their performance during the initial testing. We found that the sub-groups differed in their improvements achieved in *Picture Arrangement*, *F*(2.66) = 4.44; *p* < 0.05; *eta*^2^ = 0.12. The sub-group with the lower initial performance achieved a noticeable improvement (*p* < 0.05), whereas the gains showed by the “better” sub-group were smaller and statistically insignificant (*p* = 1). There were also some differences concerning *Block Design*, *F*(2.66) = 9.17; *p* < 0.001; *eta*^2^ = 0.22. It appeared that the level of improvement in the control group and in the “better” experimental sub-group was statistically insignificant (*p* = 1), and that it was the experimental group with lower initial results that improved substantially (*p* < 0.005). No differences were found concerning *Object Assembly*, F(2.66) = 1.89; *p* = 0.15; *eta*^2^ = 0.05. These results indicate that working memory training had the greatest impact on nonverbal reasoning among children who initially showed poor performance in a given area. In other words, improvement is more likely to occur among children who start off with lower levels of competence.

### 3.4. Delayed Testing (after 15 Months)

Thirty participants (experimental group: 18, control: 12) took part in the delayed testing. Table 4 depicts descriptive statistics pertaining to performance in two tasks at the three testing times. We found significant interactions between the group and the time of measurement in the *Gotcha!* (*F*(2.60) = 12.07; *p* < 0.001; *eta*^2^ = 0.29), and *Zoo* (*F*(2.58) = 5.99; *p* < 0.01; *eta*^2^ = 0.17) tasks, but not in the case of *Raven’s Progressive Matrices* (*F*(2.46) = 0.41; *p* = 0.61; *eta*^2^ = 0.01.

In the *Gotcha!* task the groups did not differ at the beginning (*p* = 0.98), but differences appeared just after training (*p* < 0.005) and remained significant after 15 months (*p* < 0.005). In the experimental group the improvement could still be observed after 15 months. In reference to the experimental group, the statistical significance of the difference between scores obtained during the three testing phases was *p* < 0.001 (first vs. second as well as first vs. third measurement) and *p* = 1 (second vs. third measurement). The control group improved their scores neither immediately after training (*p* = 1), nor 15 months later (*p* = 0.63). In the *Zoo* task the groups differed at each testing point after training (*p* < 0.01), but not before training (*p* = 0.52). In the experimental group the improvement could still be observed after 15 months: the statistical significance of the difference between scores in the three measurements was *p* < 0.01 (first vs. second as well as first vs. third measurement) and *p* = 1.0 (second vs. third measurement). In the control group, none of the measurements following the training indicated any improvement in comparison to the first measurement (all *p* values equaled 1.0). These results suggest that the practice effects, measured with the task used during the training phase, persisted over the 15-month period of delay.

We also found interesting results of the delayed testing in the three WISC-R subtests. The effect of interaction between group and testing time proved to be statistically significant only in reference to the *Digit Span* test (*F*(2.60) = 10.38; *p* < 0.001; *eta*^2^ = 0.26), but not in the case of *Similarities* (*F*(2.37) = 1.46; *p* = 0.24; *eta*^2^ = 0.05) or *Vocabulary* (*F*(2.60) = 1.33; *p* = 0.27; *eta*^2^ = 0.04). In *Digit Span* the experimental group did better than the control one only at the second testing (*p* < 0.001), but not in the first and third testing (*p* = 0.62 and *p* = 0.55, respectively). Additionally, the performance in the experimental group improved in the second testing (*p* < 0.001); in the third measurement it declined a bit (*p* < 0.05), but it was still better than in the first testing (*p* < 0.05). In the control group there were no significant changes between the first and the second testing (*p* = 1.00) and the change observed at the third testing was marginally significant (*p* = 0.056 for the first vs. third measurements and *p* = 0.11 for the second vs. third measurements). Since *Digit Span* allows assessment of working memory capacity, these results suggest that the near-transfer effects had disappeared after the 15-month delay period.

## 4. Discussion

We arranged computerized, adaptive working memory training for schoolchildren and measured its efficiency in terms of practice effects (trained skills), near-transfer effects (working memory capacity), and far-transfer effects (intelligence). We found significant practice effects, which persisted for 15 months after the termination of training. We also found near-transfer effects concerning WMC, measured with OSPAN. We were unable to repeat the OSPAN procedure in the delayed measurement (15 months after the training); however, the WISC-R’s *Digit Span* sub-test, which also pertains to working memory processes, did not show any stability of improvement. As for intelligence, we did not find any far-transfer effects. Raven’s Matrices did not show any training-related improvement, and the observed changes may be interpreted as resulting from mere repetition of measurement. WISC-R showed improvement either in the sub-tests pertaining to working memory processes (*Digit Span*, *Arithmetic*, *Coding*) or in the subtests that probably benefitted from repeated measurement (*Vocabulary*, *Similarities*). Therefore, we conclude that our study proved the effectiveness of working memory training for working memory itself, but not for children’s fluid or crystallized intelligence.

The results pertaining to improvement in training games are congruent with the effects discussed in other studies [17,18,23,29,30,44,50,69]. Results from the delayed measurement confirm the stability of these effects, which has rarely been demonstrated in previous studies. However, we should not exclude the possibility that these effects stemmed from a change of strategy in doing the practiced tasks, rather than from an improvement of the function of WM updating. It has been showed [70] that high- and low-span people differ in terms of the strategy they adopt to deal with WM tasks. It is therefore maintained that at least some of the effects of WM training stems from the adoption of efficient mnemonic strategies, such as grouping, chunking, or chaining, rather than from incremental changes of WM capacity [71]. However, to make stronger claims we would need additional procedures, such as including control training that would consist of the same type of influence but without the increasing level of difficulty, or the use of varied tasks to estimate the effectiveness of WMU.

As for the near-transfer effects, the hypothesis that working memory training improves working memory capacity (WMC), as measured with independent tests, has been confirmed. We found that WMC, measured with the OSPAN task, improved after the training only in the experimental group. We assume that the training improved WMC, understood as the general, domain-independent skill that manifests itself in execution of tasks engaging complex cognitive functions. Other researchers [19,29,35,49,51,72] have indicated such a possibility in previous studies, but there was little evidence that WMU training might have any wider, far-transfer effects. For most cases, training of executive functions led to transfer only in terms of storage function but not processing capability [20,44], or failed to cause any increase in the scope of working memory whatsoever [19,22,39].

As for the far-transfer effects on fluid intelligence, our results seem rather discouraging. On the one hand, we observed a slight increase in the performance in Raven’s Progressive Matrices (RPM) in the experimental group, but not in the control group. On the other hand, the amount of increase was comparable in the two groups and the overall interaction effect did not reach statistical significance. So, we conclude that a training-related increase in fluid intelligence did not occur, which is a finding consistent with other studies with adults [3,18,19,24,25,32,39,41,42,73,74,75,76] and children [19,26,28,29,39,48,49]. This conclusion does not suggest that the role of working memory in determining fluid intelligence is negligible. Correlations between the scores we found in OSPAN and RPM were similar to those observed in other studies (from 0.37 to 0.60), so we suspect that the lack of far-transfer effects can be traced back to reduced plasticity of cognitive processes underlying fluid intelligence, conversely to those connected with working memory.

Regarding the WISC-R scores, children from the experimental group improved their performance in *Digit Span*, a task implicated mostly in the capacity of verbal short-term memory. Visual short-term memory (the *Coding* subscale) also benefitted from training. These effects indicate that the training influenced general working memory capacity, which was expressed not only in the domain-independent task (OSPAN), but also in domain-specific tasks (*Digit Span*, *Coding*). Notably, the experimental group achieved lower scores in *Digit Span* in the delayed testing (after 15 months) in comparison to testing immediately after training. Performance was still better than at the initial measurement; however, because similar changes were observed in the control group, this effect could have more to do with developmental processes rather than with training effects.

Performance in the *Coding* subscale, similarly to *Object Assembly* and *Arithmetic*, depends on processing speed [77,78]. Since there were increments in all tests requiring speed of processing, we assume that the training increased the basic level of mental velocity. This effect has been previously observed in some studies on WM training in adults [35,44]. However, other training studies did not reveal this transfer [19,39,79], so it probably depends on the specific characteristics of the training procedures.

WMU training had no effect on the scores in *Picture Completion*, so either there was no effect on perceptiveness or no effect on long-term visual memory [77,78]. Also, we did not observe any training-related changes in long-term verbal memory, assessed with the *Information* subtest. This result is congruent with the results of other studies, carried-out with adults, where no transfer effect on long-term memory was found [39,80]. However, a transfer of this kind has also been reported [45]. It is possible that some WISC-R subtests were not sensitive enough to capture subtle changes that could stem from the WMU training.

Improvement in *Picture Arrangement* was similar in both groups, but the experimental group started at a higher level of competence. The training failed to have an effect on *Object Assembly*. We conclude that the training was unable to influence nonverbal reasoning, especially in the case of organization of perception and causal reasoning [77,78]. Despite numerous reports on correlations between nonverbal reasoning and working memory [81], there are possibly substantial limitations in terms of the improvement of higher cognitive functions. Improvements in *Block Design* suggest that such abilities were enhanced for abstract reasoning, but this effect can be explained, at least to some extent, by the increase in processing speed.

We observed significant improvements in the *Arithmetic* test only in the experimental group. The scores in this test are believed to reflect mathematical skills, which require mental arithmetic. It must be underscored that mental arithmetic is the ability to conduct counting operations with the use of working memory [13,82]. So, we conclude that the positive effect of training on the *Arithmetic* subtest can be accounted for in terms of WMC improvement. Such a conclusion is consistent with the results obtained with the OSPAN test. Notably, improvements in the area of mathematics were also observed after other training procedures [48,49,72], including strategy training [31,83].

Now we discuss the strongest effects obtained in the study, which have rarely been observed in previous research on WM training. Improvements in verbal sub-tests *Arithmetic, Similarities* and *Vocabulary* were found only in the experimental group, suggesting enhancement of participants’ verbal reasoning [77,78]. The effects of cognitive training on verbal skills, expressed in growth of verbal and categorical fluency, have also been demonstrated in studies with a different set of training tasks which nevertheless engage the function of working memory updating (WMU) [19,39]. Studies also report significant improvements in the *Vocabulary* sub-test resulting from training based on a complex span procedure [72], although such an effect is not always significant [48,49]. WM training among typically developing children has also been reported to improve verbal information processing expressed in reading comprehension [84] or reading skills [85], and to facilitate the effectiveness of other methods aimed at developing language skills [86]. The improvement of verbal reasoning after WM training confirms the associations between WM and language understanding [9,87] or sophistication of vocabulary [88]. It also explains how working memory influences academic performance in native language curriculum assessment tests [13,16]. However, these effects can also be accounted for in terms of gains stemming from repeated measurement. The *Vocabulary* sub-test required definition of words and was scored on the basis of the completeness of the definition provided by a test subject. A full, complex and abstract definition is awarded two points, whereas an “awkward” or an insufficiently complete definition gets only one point. It seems possible that the first approach to the *Vocabulary* subtest allows schoolchildren to produce “one-point” definitions, whereas the repeated approach triggers some reasoning processes that may lead to extended, more elaborate “two-point” definitions. Similarly, in the *Similarities* sub-test participants might have benefitted from the second measurement because, during the re-test, they had a chance to replace simple one-point answers with more elaborate and abstract ones, thus resulting in higher scoring. Such an interpretation also seems justified by the fact that the third measurement, after 15 months, demonstrated even higher results than the second one, with no significant between-group differences (Table 4). Such a change did not appear in reference to any other WISC-R subtest. Hence, our results are probably better explained in terms of the test–retest effect than in terms of the beneficial effects of WM training on WISC-R results.

What is the added value of our study in comparison with other attempts to investigate the effectiveness of working memory training? We believe that the most important issue relates to the decision to administer two major tests of general intelligence, one providing a single integrated score (RPM) and the other allowing detailed assessment of the profile of intellectual abilities (WISC-R). The former did not bring about any convincing arguments concerning the far-transfer effects of WM training, whereas the latter initially suggested that not only Verbal and Nonverbal scores, but also the Full Scale might be improved due to training. However, detailed analysis of the subscales revealed that the significant training-related improvements could be easily accounted for in terms of either working memory capacity (*Digit Span, Arithmetic, Coding*) or the test-retest effects (*Vocabulary, Similarities*). In other words, WISC-R’s subscales pertaining to working memory processes (i.e., *Digit Span, Arithmetic, Coding*) corroborated the near-transfer effect that we formerly observed with OSPAN, rather than the (ostensibly) far-transfer effect that could relate to general intelligence measured with WISC-R. Had we relied solely on the general IQ measures provided by the Full Scale and the Verbal and Nonverbal scales, we could have adopted the false conclusion that our training procedures resulted in far-transfer effects, that is, that they improved children’s general intelligence. The same false conclusion could have been drawn if we had not analyzed the training-related effects of the *Vocabulary* and *Similarities* subscales. Since scores in these subscales are particularly prone to the mere repetition of testing, these results revealed artifactual test–retest effects, rather than far-transfer effects. Since the criterion tasks in many training studies usually suffer from the problem of “impurity”, i.e., they pertain to many aspects of cognition, such a detailed analysis of results seems advisable for future research in this field.

However, we cannot exclude the possibility that increasing the number of training sessions would result in significant far-transfer effects on intelligence. In comparable studies [30,54] the number of training sessions was 20 or 15, respectively. Moreover, in Wang et al.’s [54] study the training was spread across two, five, 10 or 20 days and only the greatest amount of spacing brought about significant far-transfer effects on intelligence. In our study, the number of training sessions was 10, plus two pre-test and post-test sessions consisting of the same tasks. However, our participants trained for longer in every session (about 40 min), whereas in the cited studies every session lasted 15–20 min. So, the total training time of our participants (400 min) was longer or equal to the total training time in the cited studies. It is possible, though, that the number of training sessions rather than the total training time is an important factor that determines the effectiveness of the training intervention.

We believe that our study brings about some other elements of added value. A delayed testing session is not a standard solution in training studies because it may be costly and logistically difficult. Thanks to such a methodological solution, we were able to demonstrate that, even though near-transfer effects occurred, they vanished after the 15-month period of delay. Only the practice effects persisted after the delay, which seems to be an important conclusion from a practical point of view.

Finally, let us discuss some methodological drawbacks of this study. The most important issue pertains to lack of randomization, which might have resulted in significant biases concerning the training-related changes in performance. The lack of randomization was caused by practical reasons: this was the only way to win parents’ consent and cooperation. However, we believe that lack of randomization does not undermine our conclusions. Although random assignment to experimental and control groups is a “golden rule” in methodology, it does not prevent differences between groups in the initial testing, therefore it is advisable to consider other solutions to prevent possible biases [89]. In our study, we deliberately recruited a relatively large sample of participants in order to be able to match the control group with a fraction of the experimental group. We did so in respect to both Raven’s matrices and some WISC-R subscales. Division of the experimental group at the median point resulted in perfectly matched clusters of participants who did not differ in the pre-test. In this way, we were able to find arguments for the lack of far-transfer effects caused by our training procedures.

## 5. Conclusions

Altogether, our study implies that working memory training for schoolchildren improved their working memory capacity, as measured with independent tasks. However, such training did not improve general fluid or crystallized intelligence. It seems that general mental abilities are not as malleable as specific ones. Considering the importance of working memory processes for school performance and academic achievement, such a conclusion does not seem pessimistic from an educational perspective. Unfortunately, the transfer effects did not persist over time. This finding suggests that working memory training needs systematic effort to be stable enough, as is also the case with physical training.

## Figures and Tables

**Table 1:** Mean raw scores obtained in four training games by two groups (experimental and control) at two testing points (before and after training). Standard deviations are presented in parentheses.

The Training Task	Before Training		After Training	
	EXP	CTRL	EXP	CTRL
*Sausage Dog*	73.16	70.66	91.34	74.11
	(10.68)	(18.25)	(4.77)	(10.78)
*Big Tidy-up*	78.17	71.59	80.76	75.53
	(11.10)	(12.71)	(4.84)	(12.34)
*Gotcha!*	0.31	−2.22	10.40	−1.48
	(5.44)	(7.52)	(1.90)	(7.11)
*Zoo*	−1.84	−2.56	3.69	−2.56
	(4.50)	(7.31)	(2.33)	(5.27)

Note: EXP = experimental group; CTRL = control group.

**Table 2:** Mean raw scores obtained in the OSPAN task for each level of difficulty by two groups (experimental and control) at two testing points (before and after training). Standard deviations are presented in parentheses.

OSPAN Condition	Before Training		After Training	
	EXP	CTRL	EXP	CTRL
OSPAN full	6.27	5.67	9.56	4.30
	(4.24)	(3.5)	(4.56)	(2.83)
Sequences:				
2-element	2.95	3.04	3.88	2.44
	(0.25)	(0.31)	(0.1)	(0.26)
3-element	2.27	1.85	2.93	1.51
	(0.25)	(0.31)	(0.22)	(0.27)
4-element	0.88	0.59	2.10	0.26
	(0.19)	(0.24)	(0.22)	(0.27)
5-element	0.17	0.19	0.66	0.07
	(0.11)	(0.13)	(0.14)	(0.18)

Note: EXP = experimental group; CTRL = control group.

**Table 3:** Mean raw scores in WISC-R full scale, Verbal and Nonverbal scales, and 10 subtests, obtained by two groups (experimental and control) at two testing points (pre-test and post-test). Standard deviations are presented in parentheses.

WISC-R Measure	Pre-Test		Post-Test	
	EXP	CTRL	EXP	CTRL
WISC-R full scale	202.10	191.22	241.90	207.37
	(25.04)	(29.76)	(25.30)	(32.09)
Verbal Scale	67.10	67.25	81.74	67.37
	(12.32)	(12.97)	(13.84)	(14.55)
*Information*	11.67	10.44	13.05	11.30
	(2.69)	(3.17)	(3.18)	(3.90)
*Similarities*	12.10	12.41	14.95	12.81
	(4.25)	(2.76)	(3.70)	(2.98)
*Arithmetic*	10.88	10.67	12.12	10.44
	(1.63)	(2.30)	(1.93)	(2.26)
*Vocabulary*	23.98	25.48	29.52	24.74
	(5.99)	(6.17)	(7.66)	(6.14)
*Digit Span*	8.48	8.26	12.10	8.07
	(1.76)	(1.83)	(2.63)	(2.06)
Nonverbal Scale	135.00	123.96	160.17	140.00
	(17.34)	(20.64)	(17.14)	(21.83)
*Picture Compl.*	17.21	16.96	18.43	17.59
	(2.31)	(2.26)	(2.03)	(2.81)
*Picture Arr.*	28.88	26.19	33.79	29.67
	(5.70)	(4.94)	(4.95)	(6.97)
*Block Design*	21.31	18.59	24.98	20.37
	(5.13)	(4.03)	(5.02)	(3.85)
*Object Ass.*	29.40	26.11	35.76	31.26
	(6.39)	(7.17)	(5.57)	(6.57)
*Coding*	38.19	36.11	47.21	41.11
	(7.46)	(8.50)	(9.06)	(8.98)

Note: EXP = experimental group; CTRL = control group; Picture Compl. = Picture Completion; Picture Arr. = Picture Arrangement; Object Ass. = Object Assembly.

**Table 4:** The comparison of mean raw scores obtained by two groups (experimental and control) at three testing points (before training, immediately after training, 15 months after training). Standard deviations are presented in parentheses.

Dependent Variable	Pre-Test	Post-Test	Delayed Post-Test
*Gotcha!*			
EXP	0.44 (6.07)	10.67 (1.75)	9.97 (2.14)
CTRL	0.57 (7.54)	0.14 (7.24)	2.86 (8.32)
*Zoo*			
EXP	−2.06 (1.16)	3.77 (0.87)	4.24 (1.18)
CTRL	−0.93 (1.28)	−1.29 (0.96)	−0.64 (1.29)
*Raven*			
EXP	34.44 (9.14)	35.28 (8.97)	40.11 (6.71)
CTRL	30.21 (7.34)	31.07 (7.56)	34.00 (5.75)
*Similarities*			
EXP	12.50 (0.93)	14.83 (0.79)	18.17 (0.98)
CTRL	13.00 (1.06)	13.29 (0.89)	16.64 (1.11)
*Vocabulary*			
EXP	24.61 (6.62)	27.83 (8.38)	33.00 (9.34)
CTRL	26.21 (6.04)	26.50 (6.32)	34.57 (6.32)
*Digit Span*			
EXP	8.78 (2.07)	12.61 (3.09)	10.72 (2.54)
CTRL	8.43 (1.83)	8.43 (2.17)	10.14 (2.93)

Note: EXP = experimental group; CTRL = control group. Remark: Figures concerning pre-test and post-test differ slightly between Table 1 and Table 3 because some participants dropped out from the third phase of testing.

## Appendix A. Description of the Training Tasks

### Appendix A.1. Sausage Dog (Picture Completion)

In the *Sausage Dog* task, containers with pictures of objects divided into pieces were displayed. The number of objects, as well as the number of containers, ranged from two to five. The drawings mostly showed animals, vehicles, foods, or everyday objects. The objects represented separate categories and the pieces were just examples of these categories. The task was to put the pieces together from the fragments displayed so as to reconstruct the drawing. Only one piece was displayed at once. By pressing arrows on the keyboard, the player had to decide whether to throw the piece into the container with the object (if it fitted), or whether to discard it (if it did not fit). The objects had to be put together starting from the left side. When the object was complete, it had to be put together again. For example, if the player was putting together a dog from the (1) head; (2) body; (3) rear with the tail, that player should have first chosen piece 1, then 2, then 3, and then start putting the object together again. After an incorrect reaction, the player had to start putting the particular object together from the beginning. Each attempt took three minutes, then feedback was given, and the level of difficulty was adjusted. The game consisted of 16 stages and the rehearsal; the objects were different in each stage. The easiest required putting together two objects, i.e., each object was divided into two parts. The most difficult stage required putting together five objects, i.e., each object was divided into five parts. As the difficulty level increased, first the number of elements that the object was divided into grew, and only after that did the number of objects (categories) increase. The local feedback was provided in such a way that the container with the object was highlighted in green after a correct reaction and in red after an incorrect one. Also, an unpleasant sound accompanied the red color and the participant was informed about the termination of the current attempt. The global feedback consisted of a continuous display of the number of collected points. After each attempt the screen showed the number of points and informed the participant if he/she had completed the task correctly.

### Appendix A.2. The Big Tidy-Up (Object Arrangement)

In the second game, based on the mental tracking paradigm, the screen displayed containers (furniture pieces) where various objects are usually stored. There were five containers available: fridge, bookcase, mirror cabinet, shoe cabinet, and a pet store shelf. Each piece of furniture had five shelves on which various items could be put. Depending on the level of difficulty, there were from two to five pieces of furniture displayed with two to five items on their shelves. The shelves were always to be stacked from bottom to top in the predefined sequence of items. For example, the products in the fridge always started from the lettuce on the bottom shelf. Then, the remaining shelves had to be filled in with milk, mushrooms, tomato sauce, and carrots. Consecutive items were displayed above the containers and the player’s task was to decide (using arrows on the keyboard) if the item should be placed on the shelf at that particular time or not. It was important that the items were placed in the predetermined order from the bottom shelf to the top one. If the child made a mistake, the stacking of shelves in the current furniture piece had to be restarted from the beginning. Each attempt lasted three minutes, after which the child was given feedback and the difficulty level was adjusted. The game consisted of sixteen attempts and a rehearsal. The easiest stage required putting two objects into two containers; the most difficult required putting five objects into five containers. As the level of difficulty increased, first the number of objects went up, and then the number of containers increased. Local and global feedback was provided as in the previously described game. After each attempt, the screen showed the number of points and informed the child if the task had been completed correctly.

### Appendix A.3. Gotcha! (Catching the Thief)

This game was based on a story about a residential area that needed to be protected against a thief. The computer screen displayed a residential area divided into 16 parts, representing separate plots of land. Depending on the setting, there were from one to 16 houses, but only one in a plot. A drawing of a thief could appear by any of the houses. If the thief appeared for the second time by the same house, the player had to react by pressing the spacebar. Depending on the setting, the thief could reappear at the same house after a predetermined n-number of all appearances. After a predetermined number of stimuli, the thief fell asleep and after the game was resumed the number of the thief’s appearances needed to be counted from the beginning. One attempt consisted of three such sub-phases divided by the thief’s “naps”, so the whole attempt lasted about three minutes. There were a maximum of 47 levels of difficulty in the game, which was determined by the number of houses (a maximum of 16) and the determined n-number (from two to five). The local feedback consisted of a color change: the street/district was highlighted in green/red after a correct/incorrect reaction. After the attempt, the screen displayed information on whether the answers had been correct or incorrect. Also, one drawing of a decorative element (e.g., a dog, a plant, or a car, a house) was added or subtracted, depending on performance. The global feedback consisted of the number of gathered points that was continuously displayed. Also, the color of the lawns by the houses changed: they became a bit greener as the game progressed and more yellow when the performance declined. After each attempt, the number of points was displayed.

### Appendix A.4. Zoo (Feeding the Animals)

In this case, the narrative required the child to play the role of a zookeeper and make sure that each animal was fed only once a day. The screen displayed a feeding trough approached by subsequent animals. When a particular animal appeared by the trough for the second time, the child needed to press the spacebar. After a while, day turned into night and then the count was reset. One attempt, lasting for about three minutes total, consisted of three sub-attempts separated by nighttime, and then the animal appearances had to be counted from the beginning. There could be up to 24 levels of difficulty: the number of animals to be fed (up to 24) and the n-number, i.e., how many other animals appeared before the animal appeared again (from two to five). In order to provide the local feedback, the trough was highlighted in green/red after a correct/incorrect reaction. Also, after the attempt the screen displayed information on whether answers were correct or incorrect and one animal was added or subtracted, depending on performance. The global feedback consisted of the number of gathered points that was continuously displayed on the screen. Also, the color of the leaves by the trough changed: they became greener as the game progressed and more yellow when the performance declined. After each attempt, the number of points was displayed.

### Appendix A.5. Control Training Tasks

Training for the control group consisted of two games designed for children aged 7–10. The games were supplied by a commercial education website to be used for scientific purposes. The games were designed in cooperation with a cognitive psychologist and were aimed at stimulating perceptual problem-solving skills. The procedure of the tasks included feedback information (number of points) given after each attempt and the level of difficulty depended on how well the child performed. Doing well meant proceeding to a more difficult level and doing poorly took the child back to a lower level. The time allowed for one attempt varied but did not exceed sixty seconds. One of the games, *Engine room*, required arranging presented objects to make a complex machine complete. The objects were varied in terms of shape and the slots in the machine were shaded. The attempt was concluded when the user had made three mistakes, or the time limit ran out. The levels of difficulty were based on the time allowed to complete a task and the number of objects that had to be fitted. The second game, *Ferreting about*, required the user to maneuver a mouse through a labyrinth, gather pieces of cheese, avoid obstacles (which temporarily stopped the movement of the mouse and returned it to the entry of the labyrinth), and deliver the gathered elements to another mouse standing at the exit of the labyrinth. The attempt was completed when the time elapsed. The subsequent phases of the game became more and more difficult because of the growing complexity of the labyrinths and increasing number of the elements.
